# Supplementary material for: Analysis of the leaf methylomes of parents and their hybrids provides new insight into hybrid vigor in Populus deltoides
Source: BMC Genet. 2014 Jun 20;15(Suppl 1):S8. doi: 10.1186/1471-2156-15-S1-S8 (PMC4118634; doi:10.1186/1471-2156-15-S1-S8)
Supplement: Additional file 1 — includes Table S1, which gives detailed information about the growth comparisons between the parents and F1 hybrids. Tree height and DBH were measured during five successive years. Means are given with ± SE. Different letters indicate significant difference (P < 0.05) among the lines in trees of the same age. [file 1471-2156-15-S1-S8-S1.doc]

**Additonal File 1**

**Table S1** **Growth comparison between the parents and F1 hybrids.**

Tree height and DBH were measured during five successive years. Means are given with ± SE. Different letters indicate significant difference (P < 0.05) among the clones in trees of the same age.

Table S1 Growth comparison between the parents and F1 hybrids.

| Clone No. | One-year | | Two-year | | Three-year | | Four-year | | Five-year | |
| --- | --- | --- | --- | --- | --- | --- | --- | --- | --- | --- |
|  | tree height (m) | DBH (cm) | tree height (m) | DBH (cm) | tree height (m) | DBH (cm) | tree height (m) | DBH (cm) | tree height (m) | DBH (cm) |
| P1 | 3.51±0.06 b | 2.53±0.07 b | 6.52±0.10 c | 6.75±0.15 cd | 9.90±0.16 b | 10.60±0.22 c | 12.31±0.11 bc | 13.45±0.55 b | 13.70±0.29 bc | 15.31±0.68 a |
| 10/17 | 4.17±0.10 a | 2.69±0.09 b | 6.77±0.15 bc | 7.11±0.27 c | 9.77±0.12 b | 11.71±0.37 b | 12.19±0.08 cd | 14.99±0.34 a | 13.11±0.40 c | 16.73±0.47 a |
| H_1_ | 4.21±0.07 a | 3.10±0.08 a | 7.24±0.15 b | 8.21±0.24 b | 10.30±0.18 b | 12.54±0.26 a | 12.65±0.12 c | 15.56±0.27 a | 14.77±0.65 a | 16.94±0.62 a |
| H_2_ | 4.14±0.07 a | 3.08±0.08 a | 7.98±0.09 a | 7.91±0.15 b | 12.07±0.09 a | 12.05±0.18 ab | 13.76±0.12 a | 15.19±0.25 a | 15.42±0.59 a | 16.98±0.60 a |
| H_3_ | 4.33±0.09 a | 3.24±0.10 a | 8.04±0.14 a | 8.44±0.19 a | 11.85±0.13 a | 12.62±0.21 a | 13.30±0.21 b | 15.20±0.27 a | 15.22±0.36 a | 16.85±0.38 a |
| L_1_ | 4.07±0.09 a | 2.56±0.13 b | 6.60±0.21 bc | 6.24±0.21 d | 10.30±0.24 b | 9.56±0.29 d | 11.96±0.16 d | 11.82±0.38 b | 12.91±0.30 c | 13.23±0.29 b |
| L_2_ | 3.63±0.08 b | 2.45±0.09 b | 6.60±0.17 bc | 6.12±0.21 d | 9.80±0.18 b | 9.71±0.29 d | 12.00±0.07 cd | 11.98±0.37 b | 12.66±0.41 c | 13.08±0.96 b |

Tree height and DBH were measured during five successive years. Means are given with ± SE. Different letters indicate significant difference (P < 0.05) among the clones in trees of the same age.
