# Supplementary figures and images for: Analysis of the leaf methylomes of parents and their hybrids provides new insight into hybrid vigor in Populus deltoides
Source: BMC Genet. 2014 Jun 20;15(Suppl 1):S8. doi: 10.1186/1471-2156-15-S1-S8 (PMC4118634; doi:10.1186/1471-2156-15-S1-S8)

### Figure S 1

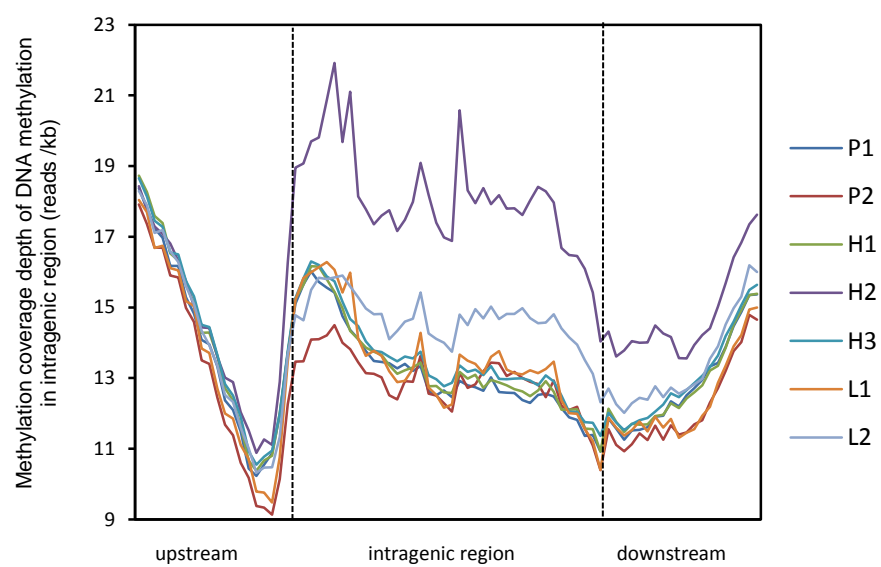

Figure S 2

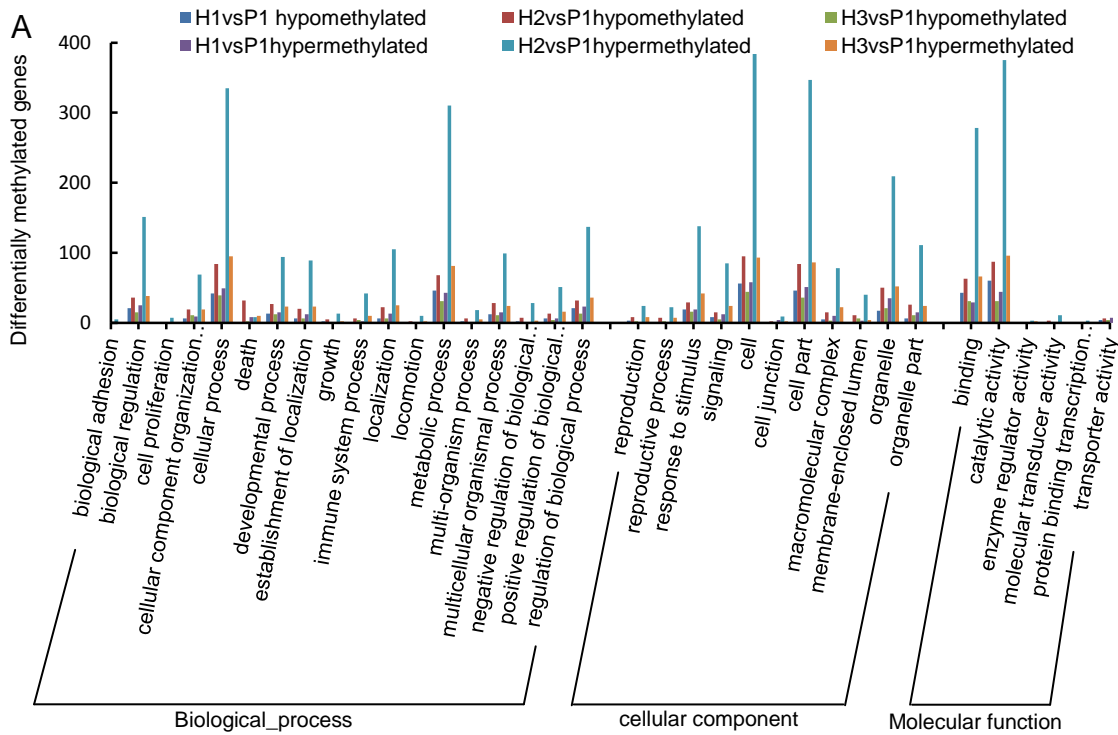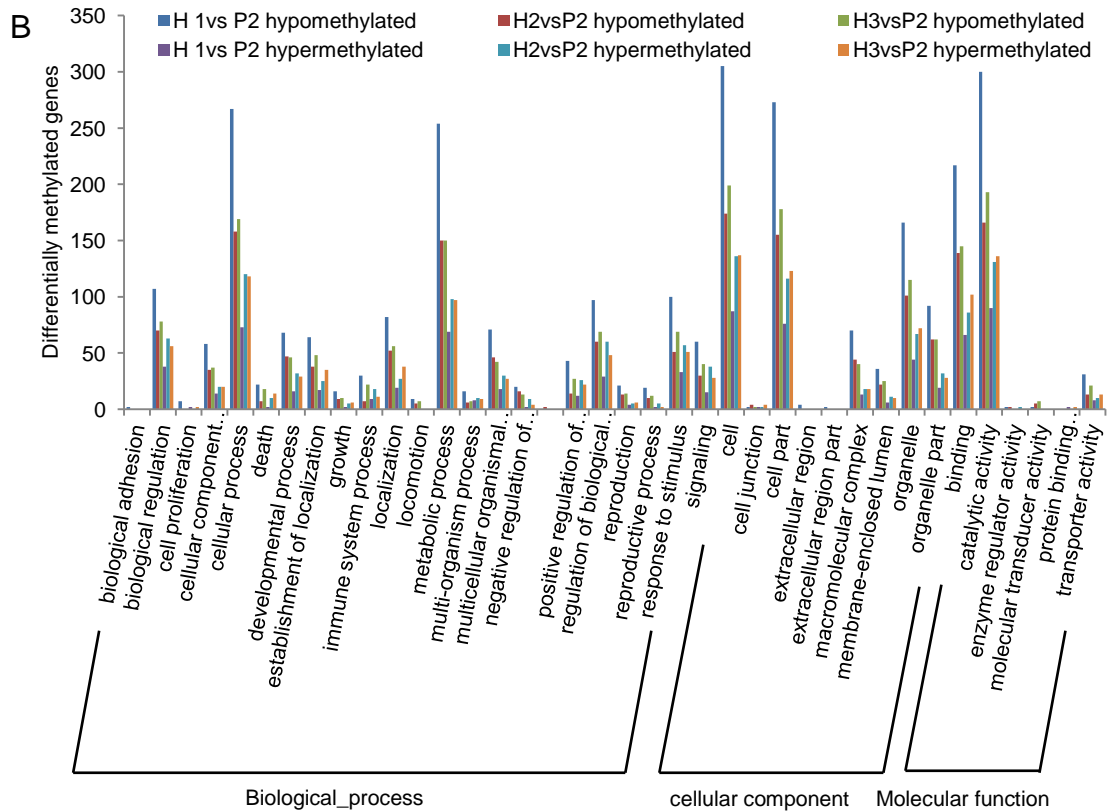

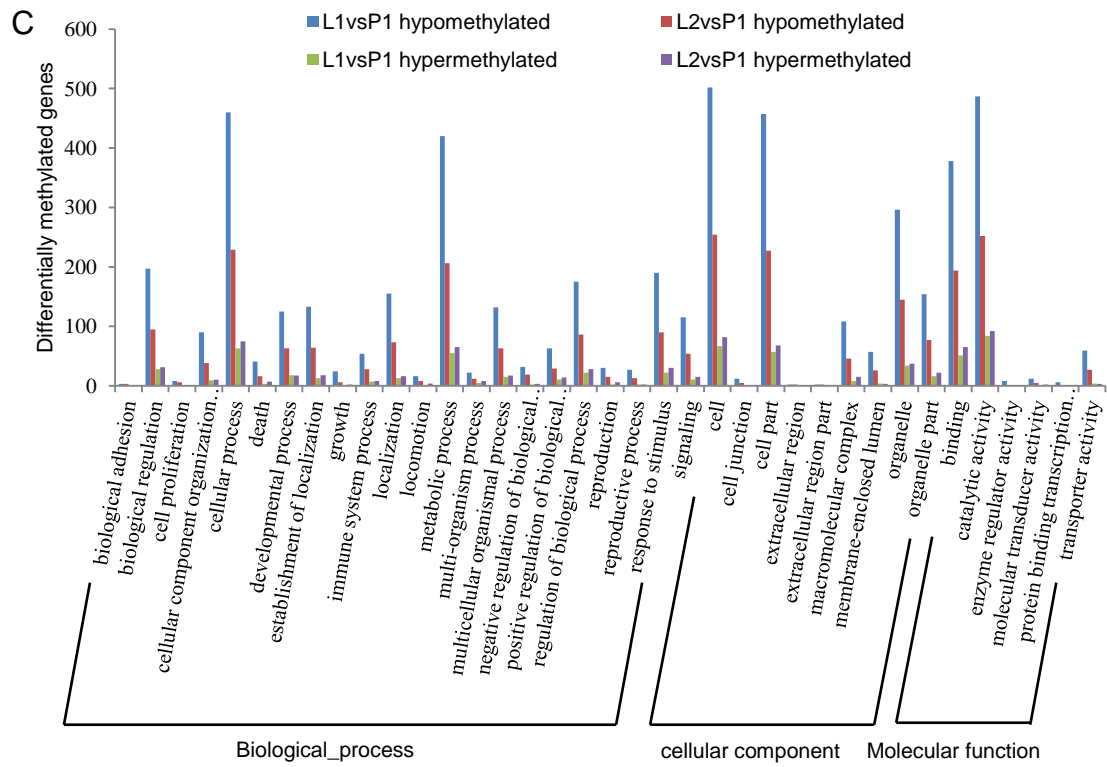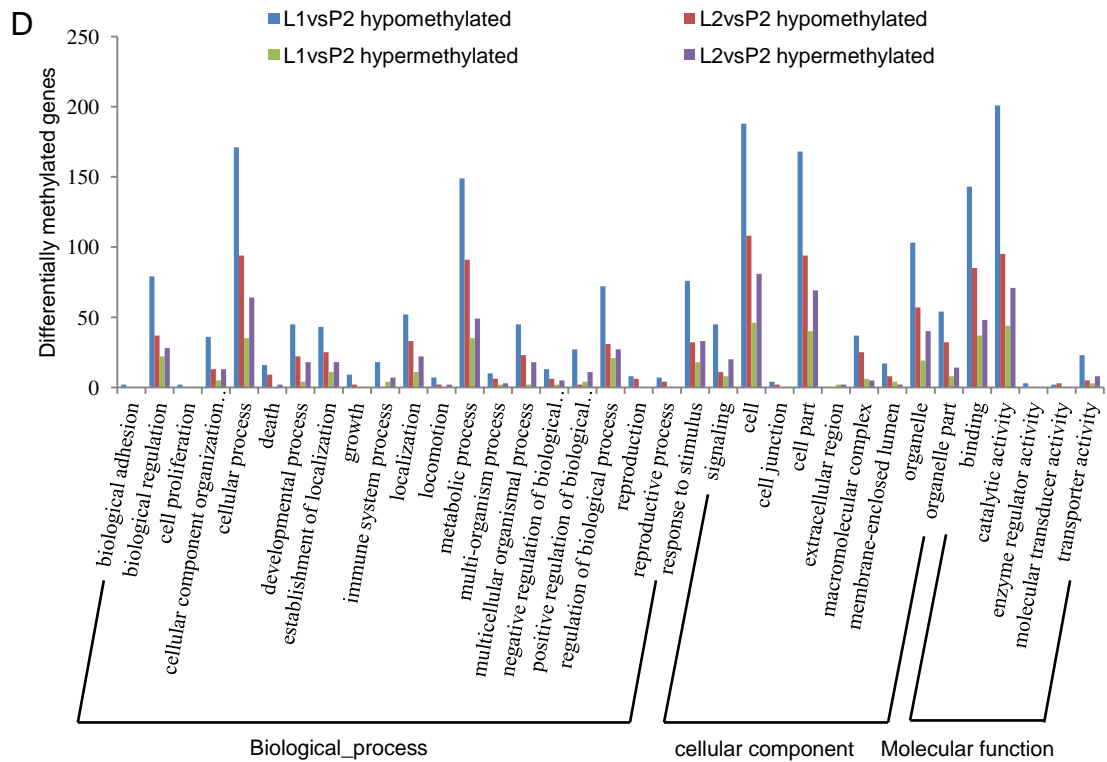

Supplement: Additional file 2 — includes Figure S1 and Figure S2. Figure S1 describes the trend of average coverage depth of DNA methylation in the intragenic region. The region within the dotted line indicates intragenic region. 2,000-bp regions upstream and downstream of intragenic are divided into 20 segments, and the intragenic are divided into 40 segments. Figure S2 provides details of GO analysis of differentially methylated genes. (A) GO analysis of differentially methylated genes between the maternal parent and better-parent F1 hybrids. Three comparison pairs (H1 versus P1, H2 versus P1, and H3 versus P1 were included in this analysis. (B) GO analysis of differentially methylated genes between the paternal parent and better-parent F1 hybrids. H1 versus P2, H2 versus P2, and H3 versus P2 were included in this analysis. (C) GO analysis of differentially methylated genes between the maternal parent and lower-parent F1 hybrids. Two comparison pairs (L1 versus P1, L2 versus P1) were included in this analysis. (D) GO analysis of differentially methylated genes between the paternal parent and lower-parent F1 hybrids. L1 versus P2 and L2 versus P2 were included in this analysis. [file 1471-2156-15-S1-S8-S2.pdf]
